# The effect of stimulation frequency on transcranial evoked potentials

**DOI:** 10.1515/tnsci-2022-0235

**Published:** 2022-08-05

**Authors:** Giorgio Leodori, Lorenzo Rocchi, Marco Mancuso, Maria Ilenia De Bartolo, Viola Baione, Matteo Costanzo, Daniele Belvisi, Antonella Conte, Giovanni Defazio, Alfredo Berardelli

**Affiliations:** IRCCS Neuromed, 86077 Pozzilli (IS), Italy; Department of Human Neurosciences, Sapienza University of Rome, 00185 Rome (RM), Italy; Department of Medical Sciences and Public Health, University of Cagliari, SS 554 bivio Sestu - 09042 Monserrato, 09124 Cagliari (CA), Italy; Institute of Neurology, University Hospital of Cagliari, Cagliari, Italy

**Keywords:** transcranial magnetic stimulation, electroencephalography, TMS–EEG, EEG, transcranial evoked potential, interstimulus interval

## Abstract

**Introduction:**

Transcranial magnetic stimulation-evoked electroencephalography potentials (TEPs) have been used to study motor cortical excitability in healthy subjects and several neurological conditions. However, optimal recording parameters for TEPs are still debated. Stimulation rates could affect TEP amplitude due to plasticity effects, thus confounding the assessment of cortical excitability. We tested whether short interpulse intervals (IPIs) affect TEP amplitude.

**Methods:**

We investigated possible changes in TEP amplitude and global mean field amplitude (GMFA) obtained with stimulation of the primary motor cortex at IPIs of 1.1–1.4 s in a group of healthy subjects.

**Results:**

We found no differences in TEP amplitude or GMFA between the first, second and last third of trials.

**Discussion:**

Short IPIs do not affect TEP size and can be used without the risk of confounding effects due to short-term plasticity.

## Abbreviations

B1, B2, B3: block 1, 2, 3
EEG: electroencephalography
EMG: electromyography
FDI: first dorsal interosseous
GMFA: global mean field amplitude
ICA: independent component analysis
IPI: interpulse interval
M1: primary motor cortex
RMT: resting motor threshold
TEP: TMS-evoked potential
TMS: transcranial magnetic stimulation
ToI: time window of interest

## 1 Introduction

The combination of transcranial magnetic stimulation (TMS) and electroencephalography (EEG) has become an increasingly used method to assess cortical physiology in the intact human. Advantages over other brain stimulation techniques include the possibility to stimulate different superficial cortical areas [1,2,3] and to extract a large number of metrics to assess cortical dynamics, including time [4,5] and time/frequency [5,6] domain measures, as well as multiple indexes to assess cortico-cortical connectivity [7,8]. The transcranial evoked potential (TEP) represents the time-domain average of TMS–EEG signals. Although obtainable by multiple cortical areas, the TEP measured by stimulation of the primary motor area (M1) is particularly robust and its features can provide useful information on cortical excitability in healthy subjects [9] and patients with movement disorders [10,11,12]. When TMS is delivered over M1, characteristic TEP components are obtained, which are named according to their polarity (positive, P; negative, N) and latency in ms: N15, P25/P30, N45, P60/P70, N100 and P180 [1]. Despite its use in research, the methodology to obtain TEPs is not fully standardized [13]. For instance, the interpulse interval (IPI) (i.e., the time that separates consecutive TMS pulses in a recording block) varies considerably across different studies, usually ranging from 4 s [6,14] to values close to 1 s [10,11,15]. Several TMS studies, using either motor-evoked potentials (MEPs) or TEPs as outcome variables, have suggested that a large number of TMS pulses (around 1,000) applied with an IPI as low as 1 s [low-frequency repetitive TMS (rTMS)] is capable of inducing cortical long-term depression (LTD)-like plasticity [16,17]. A decrease in MEP size has, however, been described with a number of stimuli as low as 60–120 [18], which is commonly used to obtain TEPs [4,19,20,21]. It is thus possible that an IPI close to 1 s may influence the amplitude of TEPs. Investigating this possibility is of importance to exclude within-block changes of TMS–EEG outcome variables, as well as possible carry-over effects across multiple recording blocks, and in the perspective of a standardization of methods in the TMS–EEG field [13].

To test whether short IPIs may influence TEPs, we measured their amplitude trend during a block of 100 trials delivered with IPIs jittering randomly between 1.1 and 1.4 s (0.9–0.7 Hz stimulation rate). If this stimulation rate produced inhibitory short-term plasticity changes [17,18], TEP amplitude would be expected to progressively decrease during the stimulation block.

## 2 Materials and methods

### 2.1 Participants

We enrolled 16 right-handed healthy volunteers [15 males, mean age ± standard deviation (s.d.) 30.2 ± 3.0 years]. Participants did not have history of neurological or psychiatric disorders, were not taking medications known to affect the central nervous system and did not have contraindications to TMS [22].


**Ethical approval**: The present research complied with all the relevant national regulations and institutional policies, was in accordance with the tenets of the Declaration of Helsinki, and had been approved by the authors’ institutional review board or equivalent committee.
**Informed consent**: Informed consent has been obtained from all individuals included in this study.

### 2.2 TMS and EMG

Participants were tested in a single experimental session. They were seated in a comfortable chair and were asked to relax and fixate a cross displayed on a PC screen about 90 cm in front of them. Participants wore earphones continuously playing a masking noise designed to reduce the perception of the TMS click [4,23]. The intensity of the noise was increased to a level sufficient to suppress the TMS click, or to a maximum intensity of 90 dB. A complete suppression of the TMS click was obtained in 11 participants, while a slight residual perception was reported by the remaining 5 (average visual analog scale value 0.44 ± 0.73, mean ± s.d.). Single-pulse TMS was delivered through a Super Rapid^2^ biphasic magnetic stimulator, connected to a figure-of-eight 70 mm coil (Magstim Ltd, Whitland, UK), over the spot on the scalp overlying the left primary motor cortex (M1) evoking the largest and most consistent MEP in the right first dorsal interosseous (FDI) muscle. We used a neuronavigation system (SofTaxic, EMS srl, Bologna, Italy) to monitor coil positioning throughout the experiment. We calculated the resting motor threshold (RMT) as the lowest stimulation intensity that produced a MEP of at least 50 µV in 5 out of 10 consecutive trials in the relaxed right FDI [24]. The experimental block consisted of 100 single pulses delivered at 90% RMT intensity with IPIs randomly ranging between 1.1 and 1.4 s (0.7–0.9 Hz). EMG was recorded from the right FDI muscle through a pair of Ag/AgCl surface electrodes, arranged in a belly-tendon fashion, bandpass filtered (10–1,000 Hz), amplified (×1,000) (D360, Digitimer Ltd, Welwyn Garden City, UK) and digitized at 5 kHz (CED1401; Cambridge Electronic Design, UK).

### 2.3 TMS–EEG signals’ recording and analysis

EEG was recorded from 32 passive electrodes on an elastic cap (BrainCap, Easycap GmbH, Wörthsee, Germany) according to the international 10–20 system [25], using Fpz as ground and POz as online reference. Impedance for each channel was kept below 5 kΩ. EEG signals were recorded using a TMS-compatible amplifier (NeurOne, Bittium Biosignals Ltd, Kuopio, Finland), hardware-filtered (low pass 2.5 kHz) and digitized at 5 kHz.

TMS–EEG signals’ preprocessing was performed with EEGLAB 14.1.1 [26] with the addition of some functions included in the TMS–EEG signal analyser (TESA) toolbox [27], running in MATLAB environment (Version 2017b, MathWorks Inc., Natick, USA). The analysis pipeline was similar to that used in a previous study from our group [11]. EEG was epoched from −1.1 s before to 1.1 s after TMS pulses and demeaned (whole epoch as baseline). Epochs and channels excessively contaminated by artifacts were eliminated. The signal from 5 ms before to 10 ms after TMS was cut to remove the electromagnetic pulse artifact and downsampling to 1,000 Hz was performed. A first round of independent component analysis (ICA) was done to remove the voltage decay artifact and TMS-evoked EMG due to scalp muscle activation [28]. The signal removed from −5 to 10 ms was then interpolated with a cubic function and fourth order Butterworth filters were applied (1–100 Hz bandpass and 48–52 Hz bandstop). Epochs were restricted (±1 s) to remove possible edge artifacts caused by filtering. Finally, we ran a second round of ICA to remove residual, non-TMS-locked artifacts (e.g., eyeblinks, horizontal eye movements, continuous muscle activity). The final, preprocessed TMS–EEG signal was re-referenced to a common average reference. Signals in each epoch were divided into three blocks of 30 trials each on average (range 25–33, see Results section for further details), named block 1 (B1), block 2 (B2) and block 3 (B3), with lower numbers indicating earlier epochs. By averaging trials in each block, we extracted the TEP, as the time-domain average of TMS–EEG signals, and the global mean field amplitude (GMFA), the latter based on the following formula:

{\rm{GMFA}}=\sqrt{\frac{\mathop{\sum }\limits_{i}^{k}({V}_{i}(t)-{V}_{{\rm{mean}}}({t\left))}^{2})}{K},}\hspace{1em}]

where *t* is time, *K* is the number of channels, *V*_*i*_ is the voltage in channel *i* and *V*_mean_ is the mean of the voltage in all channels [29].

Statistical analyses were performed using three time windows of interest (ToI) previously considered [4] ranging from 15 to 65 ms (ToI 1), 65 to 120 ms (ToI 2) and 120 to 270 ms (ToI 3) following the TMS pulse (analysis 1), as well as considering ToIs corresponding to the main TEP peaks ranging from 12 to 18 ms (N15), 25 to 35 ms (P25/P30), 40 to 50 ms (N45), 55 to 70 ms (P60/P70), 90 to 110 ms (N100) and 160 to 200 ms (P180) (analysis 2). TEPs in each ToI were compared between blocks at the scalp map level using cluster-based permutation testing as implemented in Fieldtrip toolbox for Matlab [30] (Monte Carlo, 5000 permutations, clusters significant for *p* < 0.05). Possible differences in the GMFA across the three blocks were investigated by means of a two-way repeated measures analysis of variance (ANOVA) with “block” (B1, B2, B3) and “ToI” (ToI 1, 2, 3) as factors of analysis (SPSS v.27, IBM Corp, Armonk, USA). Results are expressed as mean ± s.d. unless otherwise specified. Normality of distribution of the GMFA values was assessed by means of the Shapiro–Wilk’s test. GMFA values above or below mean ±3 s.d. were considered outliers and corrected by winsorization. *p* values < 0.05 were considered significant. The sphericity of GMFA value distribution was verified by Mauchly’s tests, and Greenhouse–Geisser correction was applied when necessary. Post hoc comparisons were conducted when appropriate by means of planned contrasts.

## 3 Results

**Figure 1:**
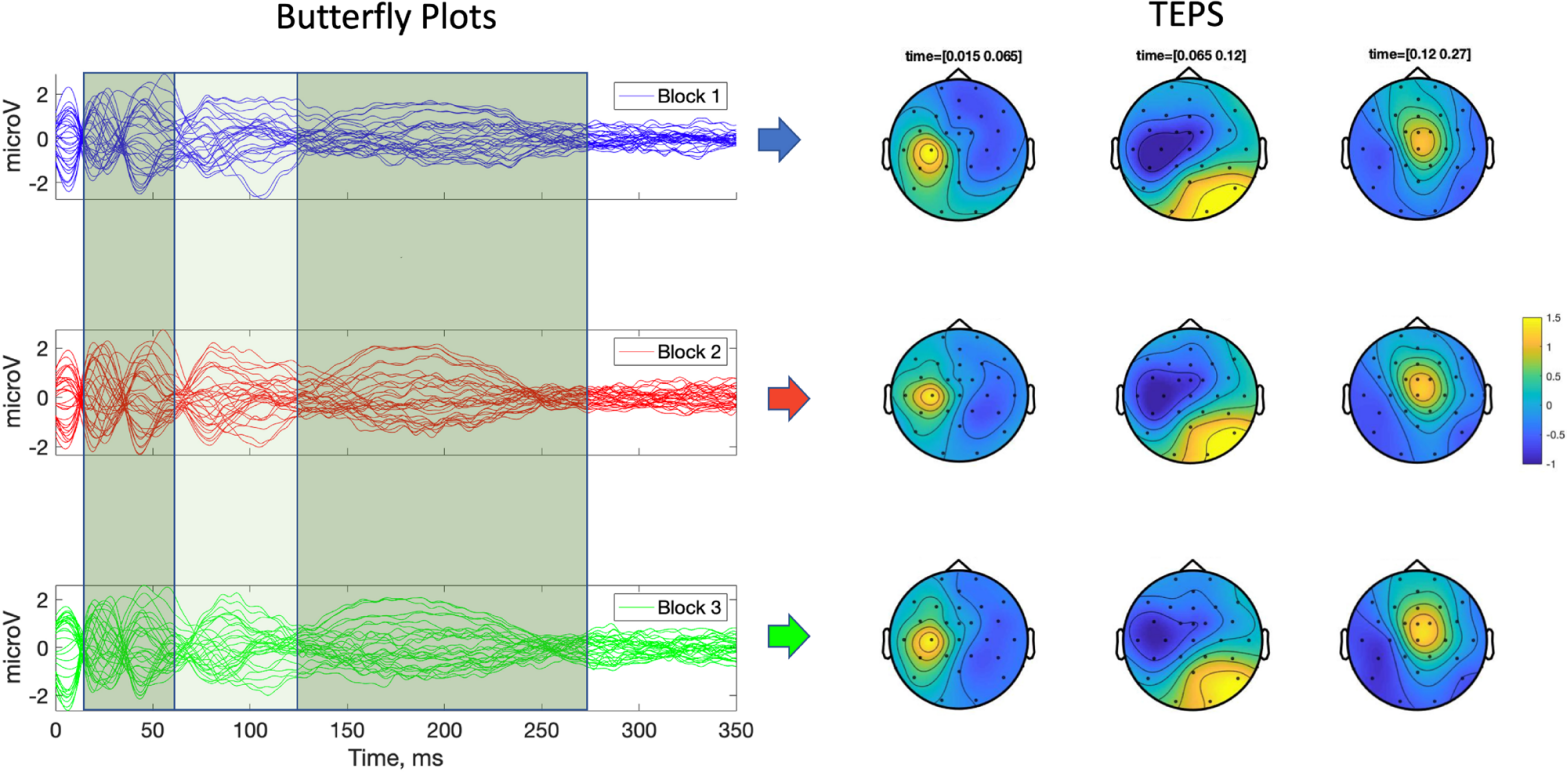
TMS-evoked potentials. (A) Butterfly plots of TEPs obtained by averaging trials in separate blocks. Colored panels indicate the time windows of interest which were used for the comparisons of TEPs. (B) Topographical plots of TEPs averaged in each time window of interest.

**Figure 2:**
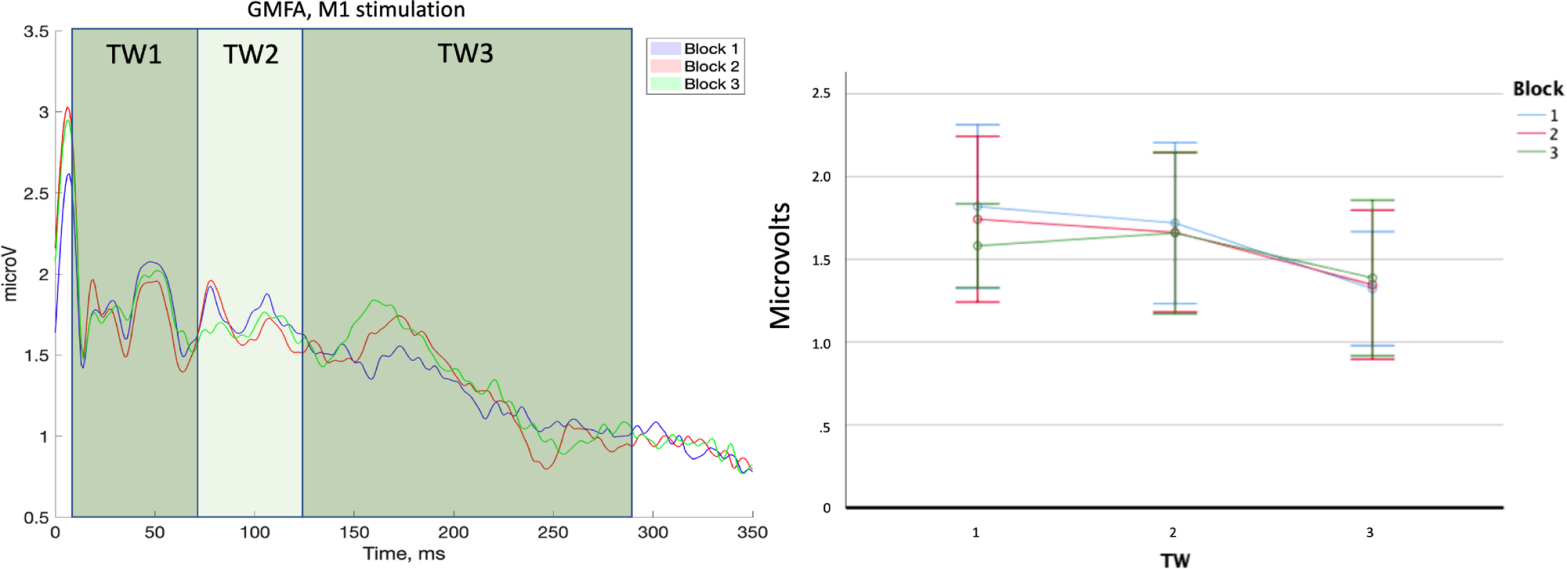
GMFA. Left panel: average GMFA across participants in the three blocks. Colored panels indicate the time windows of interest used for GMFA comparisons. Right panel: average values of GMFA in each block. TW = time window of interest. Error bars represent 2 × standard error of the mean.

All participants successfully completed the experimental procedure and no adverse effects due to TMS were observed. The mean RMT was 60.14 ± 3.70% of the maximum stimulator output. Following signal preprocessing, 32.50 (7.39), 32.63 (7.39) and 32.56 (7.39) epochs were averaged, respectively, for B1, B2 and B3. Cluster-based analysis showed no significant differences between TEPs recorded in different blocks in the ToIs considered in analysis 1 (ToI 1, 2, 3) and analysis 2 (N15, P25/P30, N45, P60/70, N100, P180) (Figure 1). GMFA values were normally distributed (no significant *p* values were found in the Shapiro–Wilk’s tests). Two significant high outliers in GMFA distributions were found and substituted through winsorization. We found no significant effect of factor “block” (*F*_2,30_ = 0.85, *p* = 0.43, η*p* ^2^ = 0.05) and “block × ToI” interaction (*F*_4,60_ = 1.18, *p* = 0.31, η*p* ^2^ = 0.07) in the ANOVA on the GMFA. We found a significant effect of factor “ToI” (*F*_2,30_ = 5.58, *p* = 0.009, η*p* ^2^ = 0.271). However, planned contrast between ToIs did not return any statistically significant differences (all *p* values > 0.05) (Figure 2).

## 4 Discussion

We tested whether short IPIs determine changes in TEP amplitude and GMFA within a recording block of 100 TMS trials delivered over M1. The results showed that TEPs and GMFA obtained by averaging three smaller blocks of around 30 TMS pulses did not significantly differ between each other. Our results, therefore, provide evidence that frequencies of 0.7–0.9 Hz can be used to stimulate M1 in the TMS–EEG context without the risk of inducing within-block effects due to repeated TMS.

Our results are in agreement with those of Julkunen and coworkers, who found that MEP amplitude remains constant during short trains of TMS [31], but may seem at odds with previous literature suggesting that long trains of repetitive TMS delivered on M1 with IPIs of 1 s produce inhibition of MEPs [16] and TEPs [17], possibly due to LTD-like effects. The latter, however, requires a number of stimuli (around 1,000) greater than that used here (100) [32,33]. Therefore, LTD-like plasticity is unlikely to have affected our results. Nojima and coworkers reported a decrease in MEP amplitude caused by rTMS delivered with IPIs and a number of pulses comparable to those used in the present study. Although the mechanism underlying this effect has not been clarified, a form of short-term synaptic plasticity is possible [18]. One factor to explain the difference with the present findings might be the nature of the outcome measure. Rather than MEPs, which reflect the net excitability of a limited circuitry within M1 [34], we assessed TEPs, which are generated by the spatial and temporal summation of excitatory and inhibitory postsynaptic potentials in a larger population of cortical neurons [1]. These responses are influenced by local excitability and by connectivity with other cortical and subcortical structures [15,35,36]. Albeit TEPs can be modulated by rTMS protocols, which are known to induce amplitude changes of MEPs [17,37], the pattern of modulation can be different [19]; this might be one reason for the lack of within-block effects in the present study. This conclusion is at least partially supported by the results obtained by Casarotto and colleagues, who, albeit not investigating within-block changes, did not find differences in amplitude of TEPs obtained with an IPI close to 1 s recorded in several sessions of the same day, thus excluding carry-over effects [38]. Another possible factor to consider is that we introduced a jitter in IPIs; by contrast, rTMS is usually performed with a fixed frequency [39]; it is, thus, possible to hypothesize that variability in rTMS frequency prevents the occurrence of cortical plasticity for short pulse trains.

We acknowledge some limitations to the present study. As residual TMS click perception was reported by 5 out of 16 participants; therefore, TEP components around 100 and 200 ms may have been partially contaminated by auditory-evoked potentials (AEPs). While we cannot provide direct evidence to rule out this possibility, a substantial contamination of our TEPs by auditory responses is ruled against by a lack of a prominent P200, characteristic of AEPs [4], and by the suppression of AEPs obtained in experimental conditions similar to the present setting [4]. In addition, AEPs show habituation, leading to amplitude decrease, with as little as 50 stimuli and an interstimulus interval close to that used here [40]; such an amplitude decrease would have been expected in our data as well if they were contaminated by AEPs.

As we used subthreshold intensity, which is the most common for TMS–EEG studies on M1 where MEPs are not investigated, we cannot exclude short-term effects on TEPs’ amplitude when higher stimulation intensities are used. In addition, TEPs in each block were obtained with a relatively small number of pulses (around 30); however, previous observations have suggested that TEPs obtained with a comparable number of trials have a high similarity to those recorded with a standard number of epochs (over 100) [20]. In conclusion, the present study provides evidence that short IPIs randomly varying between 1.1 and 1.4 s do not affect TEP size and therefore can be used to significantly reduce the duration of TMS–EEG studies without the risk of inducing signal changes related to stimulation rate.
